# Predictive value of myocardial strain on myocardial infarction size by cardiac magnetic resonance imaging in ST-segment elevation myocardial infarction with preserved left ventricular ejection fraction

**DOI:** 10.3389/fphar.2022.1015390

**Published:** 2022-10-14

**Authors:** Qiang Wang, Jian Wang, Yingjie Ma, Peng Wang, Yang Li, Jing Tian, Xiuzheng Yue, Guohai Su, Bin Li

**Affiliations:** ^1^ Department of Cardiology, Central Hospital Affiliated to Shandong First Medical University, Jinan, China; ^2^ Department of Radiology, Central Hospital Affiliated to Shandong First Medical University, Jinan, China; ^3^ Philips Healthcare, Beijing, China; ^4^ Research Center of Translational Medicine, Central Hospital Affiliated to Shandong First Medical University, Jinan, China

**Keywords:** acute ST-segment elevation myocardial infarction, preserved left ventricle ejection fraction, magnetic resonance imaging, strain, late gadolinium enhancement (LGE)

## Abstract

**Background:** The correlation between myocardial strain and infraction size by cardiac magnetic resonance imaging in ST-segment elevation myocardial infarction (STEMI) with preserved left ventricular ejection fraction (LVEF) is not clear.

**Objective:** To investigate the correlation between myocardial strain and myocardial infarction size in patients of acute STEMI with preserved LVEF.

**Materials and Methods:** A retrospective study was conducted to assess 31 patients with acute ST-segment elevation myocardial infarction (STEMI)after primary percutaneous coronary intervention (PCI) who received cardiac magnetic resonance (CMR) imaging during hospitalization at the Central Hospital of Shandong First Medical University from 2019 to 2022 and whose echocardiography indicated preserved LVEF (LVEF≥50%). The control group consisted of 21 healthy adults who underwent CMR during the same period. We compared the CMR characteristics, global and segmental strain between the two groups. Furthermore, the correlation between the global strain and the segmental strain of the left ventricle and late gadolinium enhancement (LGE) were evaluated.

**Results:** Compared with healthy controls, the left ventricular ejection fraction (LVEF) of STEMI patients with preserved LVEF was significantly decreased (*p* < 0.05). Moreover, the global radial strain (GRS) (24.09% [IQR:17.88–29.60%] vs. 39.56% [IQR:29.19–42.20%], *p* < 0.05), global circumferential strain (GCS) [−14.66% (IQR: 17.91–11.56%) vs. −19.26% (IQR: 21.03–17.73%), *p* < 0.05], and global longitudinal strains (GLS) (−8.88 ± 2.25% vs. −13.46 ± 2.63%, *p* < 0.05) were damaged in patients. Furthermore, GCS and GLS were associated with LGE size (%left ventricle) (GCS: r = 0.58, *p* < 0.05; GLS: r = 0.37, *p* < 0.05). In the multivariate model, we found that LGE size was significantly associated with GCS (β coefficient = 2.110, *p* = 0.016) but was not associated with GLS (β coefficient = −0.102, *p* = 0.900) and LVEF (β coefficient = 0.227, *p* = 0.354). The receiver operating characteristic (ROC) results showed that GCS emerged as the strongest LGE size (LGE >25%) prognosticator among strain parameters (AUC: 0.836 [95% CI, 0.692—0.981], sensitivity: 91%, specificity: 80%) and was significantly better (*p* = 0.001) than GLS [AUC: 0.761 (95% CI, 0.583—0.939), sensitivity: 64%, specificity: 85%] and LVEF [AUC: 0.673 (95% CI, 0.469—0.877), sensitivity: 73%, specificity: 70%].

**Conclusion:** Among STEMI patients with preserved LVEF after PCI, CMR-FT-derived GCS had superior diagnostic accuracy than GLS and LVEF in predicting myocardial infarction size.

##  Introduction

In the last decade, advances in reperfusion and preventive therapies have reduced ST-segment elevation myocardial infarction (STEMI)-related mortality in hospitalized patients (Szummer et al., 2017). Early revascularization is the key to saving ischemic myocardium and limiting the infarct size (Ibanez et al., 2017) and significantly reduces the incidence of adverse cardiovascular events and improves the prognosis of patients (Sugiyama et al., 2015). However, about 20% of patients experience recurrent cardiovascular events within a year following acute myocardial infarction (Pedersen et al., 2014). Thus, new assessment and diagnostic methods are necessary to develop judgments on cardiac function and infarct size and scope that are more accurate, which will assist cardiovascular physicians in making clinical decisions.

The left ventricular ejection fraction (LVEF) shown by echocardiography can reflect only global systolic function, whereas regional functional abnormalities or diastolic dysfunction cannot be accurately portrayed (Kim et al., 1999; Thiele et al., 2006). With LVEF, it is difficult to accurately determine the degree of cardiac damage and predict the infarct size (IS). Feature-tracking cardiac magnetic resonance (FT-CMR) has been developed as a more comprehensive method for measuring myocardial strain and establishing the functional status of the left ventricle, enabling the assessment of global and regional myocardial deformation (Smiseth et al., 2016). CMR examination is considered the *in vivo* reference standard for measuring infarct size in STEMI patients (Mahrholdt et al., 2005; Thiele et al., 2006). It is recommended that late gadolinium enhancement (LGE) be used for infraction size (IS) quantification (Mahrholdt et al., 2005; Thiele et al., 2006; Ibanez et al., 2019). However, data regarding the correlation between strain and LGE of FT-CMR in STEMI patients are scarce and sometimes controversial (Khan et al., 2015; Yu et al., 2021). The predictive value of strain by FT-CMR for the infarct size of STEMI patients with preserved LVEF remains unclear.

Therefore, we aimed to explore whether CMR myocardial strain could be more sensitive and accurate than LVEF in detecting the degree of cardiac dysfunction and predicting myocardial infarction size in STEMI patients with preserved LVEF. In addition, we aimed to find parameters related to the infarction size so we may better stratify patient risk in clinical practice and formulate treatment plans based on reference data.

## Materials and methods

### Study design and participants

**FIGURE 1:**
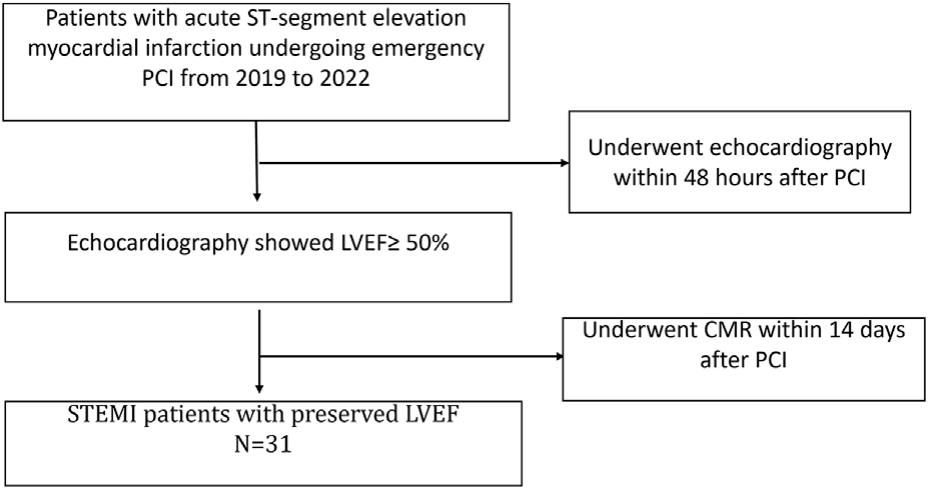
Flowchart of recruitment for patients. PCI, percutaneous coronary intervention; LVEF, left ventricular ejection fraction; CMR, Cardiac Magnetic Resonance.

The cases for this retrospective study originated from the Affiliated Central Hospital of Shandong First Medical University, which owns the first national chest pain emergency center in Jinan City. Our study selected patients who underwent emergency PCI and were hospitalized for acute ST-segment elevation myocardial infarction in the Central Hospital of Shandong First Medical University from 2019 to 2022. Thirty-one patients who underwent cardiac MRI over 14 days after PCI and whose echocardiography results showed LVEF ≥50% were selected for the study (Figure 1). Among the 31 STEMI patients with preserved left ventricular ejection fraction, 15 patients had single-vessel disease (48%), seven patients had double-vessel disease (23%), and nine patients had triple-vessel disease (29%). All these patients were admitted to our hospital with acute ST-segment elevation myocardial infarction, and we only performed revascularization of the culprit vessel during this hospitalization. Besides, all patients’ culprit vessels recovered TIMI three blood flow after PCI. In addition, patients were treated with aspirin and platelet P2Y12 receptor antagonists such as clopidogrel or ticagrelor at discharge. Twenty-one healthy adults with no history of cardiovascular disease who had undergone CMR imaging during the screening period were selected as controls.

Based on the clinical baseline characteristics of the patients and the control group, the following information was obtained: age, gender, height, weight, body mass index (BMI), body surface area (BSA), smoking status, and history of hypertension, history of diabetes and of hyperlipidemia. The following are the diagnostic criteria for ST-segment elevation (measured at the J-point) in myocardial infarction: 1) Signs and symptoms consistent with myocardial ischemia (i.e., persistent chest pain lasting more than 30 min), 2) Electrocardiogram findings (ST-segment elevation measured at the J-point) suggestive of ongoing coronary artery acute occlusion, as in the following cases: at least two contiguous leads with ST-segment elevation of 2.5 mm in men <40 years, 2 mm in men 40 years, or 1.5 mm in women in leads V2—V3 or 1 mm in the other leads [in the absence of left ventricular (LV) hypertrophy or left bundle branch block (LBBB)] or new left bundle-branch block on the 18-lead electrocardiogram and elevated troponin I level (Ibanez et al., 2017). The exclusion criteria were as follows: aortic valve disease, infiltrative disease (cardiac amyloidosis, Anderson-Fabry disease, and Danon disease), or systemic disease. Patients with MRI contraindications, such as implanted pacemakers, metallic intracranial implants, claustrophobia, and renal function impairment, were also excluded. The local ethics committee approved this study, and all subjects gave written informed consent.

### CMR imaging

Cardiac MRI was performed on 3.0 T MRI systems (Elition, Philips Healthcare, Best, the Netherlands) using a 32-channel phased-array abdomen coil. The protocol consisted of cine imaging and LGE imaging for analysis. First, standard cine images were acquired with end-expiratory breath hold steady-state free precession sequences (SSFP). Then, phase-sensitive inversion recovery (PSIR) was applied to late gadolinium enhancement (LGE) imaging. Infarct size was determined from the LGE images. The acquisitions of SSFP cine and PSIR were conducted in 2-chamber, 3-chamber, and 4-chamber long-axis planes, as well as a stack of contiguous short-axis slices, which encompassed the left ventricle from the atrioventricular ring to the apex.

Cine images were obtained using a steady-state free precession (SSFP) sequence with a breath-hold and ECG trigger for cardiac morphologic and functional analyses. The scanning parameters were as follows: repetition time (TR)/echo time (TE) = 2.8–3.0/1.4–1.5 ms, field of view (FOV) = 300 mm^2^ × 300 mm^2^, voxel = 2 mm × 2 mm × 6 mm, flip angle = 45°, and 6–8 mm slice thickness. LGE images were acquired 10–15 min after intravenous injection of 0.2 mmol/kg of gadolinium-based contrast agent (Jiangsu Hengrui Pharmaceutical Co., Ltd.) using a phase-sensitive inversion recovery (PSIR) sequence. The scanning parameters were as follows: TR/TE = 4.4/2.1 ms, FOV = 300 mm^2^ × 300 mm^2^, voxel = 1.5 mm × 1.5 mm × 10.0 mm, flip angle = 5°, and 10 mm slice thickness.

### CMR analysis

**FIGURE 2:**
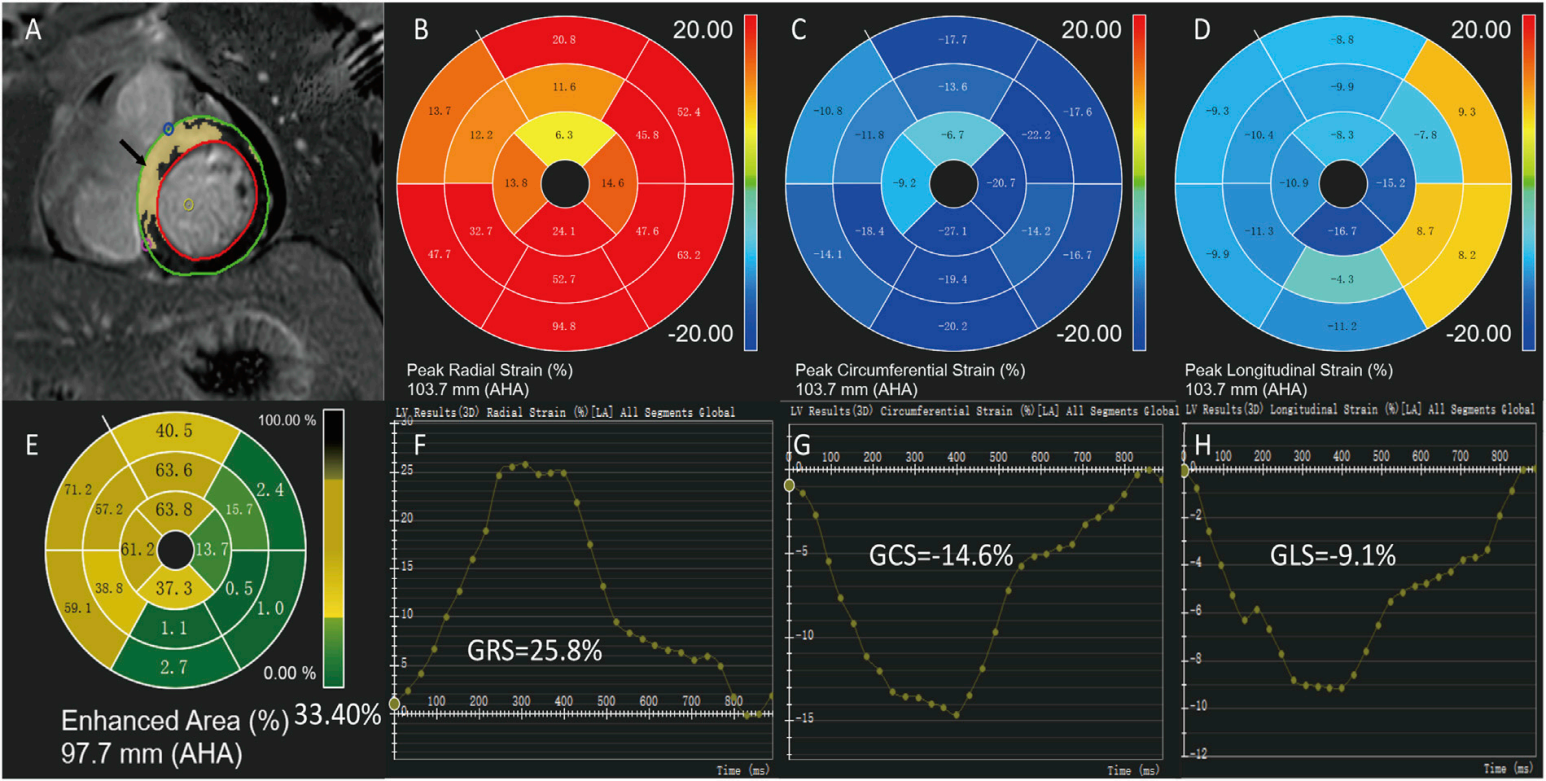
Measurement of cardiac magnetic resonance imaging. A 43-year-old male of ST-segment elevation myocardial infarction with preserved left ventricular ejection fraction. Epicardial (green) and endocardial (red) contours, LGE areas are marked in yellow **(A,E)**. Feature-tracking strain values of the same patient show global radial strain (GRS) = 25.8% **(B,F)**, global circumferential strain (GCS) = -14.6% **(C,G)** and global longitudinal strain (GLS) = -9.1% **(D,H)**.

All the analyses were conducted by two investigators with more than 5 years of experience each using the commercial software CVI42 (Circle Cardiovascular Imaging, Calgary, Canada). Endocardial and epicardial contours of the myocardium were applied, followed by subsequent software-driven automatic tracking (Figure 2). A quality adjustment was performed, and contours were amended manually, if necessary. Cardiac functional parameters were computed automatically. For quantification of contrast enhancement, the outline of the left ventricular myocardium was manually depicted, and LGE was detected by +5 SDs over the signal intensity of the normal myocardium. The LGE results were expressed as a percentage of the myocardial volume of the left ventricle (Global LGE size %). In addition, LV volumes, LV EF (LVEF), RV volumes, RV EF (RVEF), LA volumes, and LA EF (LAEF) were recorded. Feature-tracking was performed on standard long-axis cine (two, three, and four-chamber views) and short-axis cine to calculate the LV peak strain parameters, including global circumferential strain (GCS), global longitudinal strain (GLS), global radial strain (GRS), segmental peak circumferential strain, segmental peak longitudinal strain, and segmental peak radial strain (Figure 2).

### Statistical analysis

Continuous variables were presented as mean ± standard deviation (SD) or median values with interquartile range (IQR), depending on the normality variables. Student’s t-test or Mann–Whitney *U* test was applied to compare the continuous variables. Categorical variables were presented as exact numbers with percentages, and the x^2^ test or Fisher exact test was conducted for comparison. Correlation between variables was performed using Spearman’s rank or Pearson correlation test. A multiple linear regression model was constructed to assess correlates of GCS, GLS, LVEF, and LGE size. The optimal cut-off values to identify the LGE size of STEMI patients, whether higher than 25% or not, were derived from receiver operating characteristics (ROC) analysis by the Youden Index. AUCs were compared using validated methods described by DeLong et al. (DeLong et al., 1988). All tests were two-sided, and *p* < 0.05 was considered statistically significant. All statistical analyses were conducted using R version 4.1.0 (The R Foundation, Austria).

## Results

### Baseline characteristics and CMR parameters

A total of 31 STEMI patients with preserved LVEF who met the selection criteria were retrospectively identified in this study (age 51.90 ± 12.32 years; 25 men); 21 healthy controls (age 54.90 ± 12.81 years; 13 men) were also included. No differences were observed between STEMI patients and healthy controls regarding age, gender, height, weight, body mass index (BMI), and body surface area (BSA) (Table 1). In addition, the patients had a high prevalence of hypertension (51.61%), hyperlipidemia (32.26%), and diabetes mellitus (29.03%) (Table 1).

**TABLE 1:** Baseline characteristics of the study population.

Variable	STEMI (*n* = 31)	Control (*n* = 21)	*p*-value
Age, years	51.90 ± 12.32	54.90 ± 12.81	0.405
Male, n (%)	25 (80.65)	13 (61.90)	0.239
Hight, m	1.73 [1.66–1.76]	1.70 [1.65–1.74]	0.304
Weight, kg	79.06 ± 14.92	72.88 ± 9.37	0.073
Body mass index, kg/m^2^	26.12 [24.58–29.76]	24.91 [23.67–26.23]	0.119
Body surface area, m^2^	1.93 ± 0.21	1.83 ± 0.15	0.057
Hypertension, n (%)	16 (51.61)	6 (28.57)	0.173
Hyperlipidemia, n (%)	10 (32.26)	2 (9.52)	0.093
Diabetes mellitus, n (%)	9 (29.03)	2 (9.52)	0.165
Smoke, n (%)	21 (67.74)	8 (38.10)	0.068
Culprit Vessel, n (%)			-
LAD	22 (70.97)	-	
RCA	9 (29.03)	-	

LAD, left anterior descending artery; RCA, right coronary artery.

CMR examinations were conducted between the STEMI patients with preserved LVEF and healthy subjects. An overview of assessed CMR parameters is presented in Table 2. We divided the left ventricle into apical, middle, and basal parts, according to the American Heart Association model (Cerqueira et al., 2002). LGE was distributed in each part of the left ventricle. Compared with the controls, all the CMR parameters of the left ventricle, including LVEF, were impaired in the STEMI group (all *p* < 0.05). However, we did not detect any impairments of the CMR parameters of the right ventricular in the STEMI group (all *p* > 0.05).

**TABLE 2:** CMR parameters of the study population.

Variable	STEMI (*n* = 31)	Control (*n* = 21)	*p*-value
LVEF, %	49.48 ± 9.50	62.88 ± 6.96	<0.001
LV mass index, g/m^2^	58.59 [51.88–66.70]	44.59 [42.11–52.23]	<0.001
LVEDV, mL	153.99 [134.30–175.72]	131.46 [118.83–152.88]	0.013
LVEDVi, mL/m^2^	82.63 [70.51–90.83]	72.66 [66.47–77.94]	0.027
LVESV, mL	72.21 [57.20–85.53]	51.14 [37.93–58.40]	<0.001
LVESVi, mL/m^2^	37.26 [30.09–49.22]	27.11 [23.52–31.67]	<0.001
LVSVi, mL/m^2^	39.23 [33.24–44.77]	42.03 [39.62–51.18]	0.049
RVEF, %	54.66 ± 7.88	54.31 ± 7.07	0.866
RVEDV, mL	134.50 ± 31.88	139.84 ± 29.08	0.535
RVEDVi, mL/m^2^	69.85 ± 14.53	75.67 ± 12.66	0.133
RVESVi, mL/m^2^	31.74 ± 8.79	34.79 ± 8.45	0.216
RVSVi, mL/m^2^	38.24 ± 9.20	40.88 ± 7.52	0.262
Global LGE size, %	18.23 [15.55–27.87]	-	-
GLS, %	−8.88 ± 2.25	−13.46 ± 2.63	<0.001
GRS, %	24.09 [17.88–29.60]	39.56 [29.19–42.20]	<0.001
GCS, %	−14.66 [−17.91–11.56]	−19.26 [−21.03–17.73]	<0.001
Basal LS%, %	41.01 [28.03–52.97]	62.58 [45.63–78.95]	<0.001
Basal RS, %	−14.15 ± 3.68	−15.42 ± 2.94	0.173
Basal CS, %	−7.39 [−8.77–5.12]	−9.89 [−11.92–7.62]	0.012
Mid LS, %	21.56 ± 8.44	33.09 ± 9.42	<0.001
Mid RS, %	−14.89 ± 3.35	−19.43 ± 2.31	<0.001
Mid CS, %	−8.91 ± 3.11	−13.52 ± 2.78	<0.001
Apical LS, %	18.26 [9.63–24.11]	27.34 [21.47–35.16]	0.003
Apical RS, %	−16.32 ± 4.03	−22.15 ± 2.64	<0.001
Apical CS, %	−11.77 ± 2.78	−17.08 ± 2.28	<0.001
LA size, mm	69.27 ± 19.33	62.79 ± 15.32	0.185
LAV _pre-a_, mL	50.16 [39.49–60.29]	39.39 [33.68–48.14]	0.023
LAEF _total_, %	53.42 ± 9.31	58.93 ± 5.78	0.011

LVEF, left ventricular ejection fraction; LV, left ventricular; LVEDVi, left ventricular end-diastolic volume index; LVESVi, left ventricular end-systolic volume index; LVSVi, left ventricular stroke volume index; RVEF, right ventricular ejection fraction; RVEDVi, right ventricular end-diastolic volume index; RVESVi, right ventricular end-systolic volume index; RVSVi, right ventricular stroke volume index; LGE, late gadolinium enhancement; GRS, global peak radial strain; GCS, global peak circumferential strain; GLS, global peak longitudinal strain; RS, radial strain; CS, circumferential strain; LS, longitudinal strain; LA, left atrium; LAV_pre-a_, left atrial active pre-systolic volume; LAEF_total_, left atrial total ejection fraction.

Global LGE size was 18.23% [IQR: 15.55–27.87] in the patients. GRS, GCS, and GLS were significantly lower in the STEMI patients than healthy controls (GRS: 24.09% [IQR: 17.88–29.60] vs 39.56% [IQR: 29.19–42.20], *p* < 0.001; GCS: 14.66% [IQR: 17.91–11.56] vs. −19.26% [IQR: 21.03–17.73], *p* < 0.001; GLS: 8.88 ± 2.25% vs. −13.46 ± 2.63%, *p* < 0.001). Furthermore, we divided the left ventricle into apical, middle, and basal parts, according to the American Heart Association model (Cerqueira et al., 2002). The segmental strain parameters (basal strain, mid strain, and apical strain) were impaired in patients with STEMI (*p* < 0.05) except basal radial strain (*p* = 0.173). In addition, the left atrial total ejection fraction of the STEMI patients was worse than in healthy controls (*p* < 0.05) (Table 2).

### Association between LV strain, LVEF, and LGE

**FIGURE 3:**
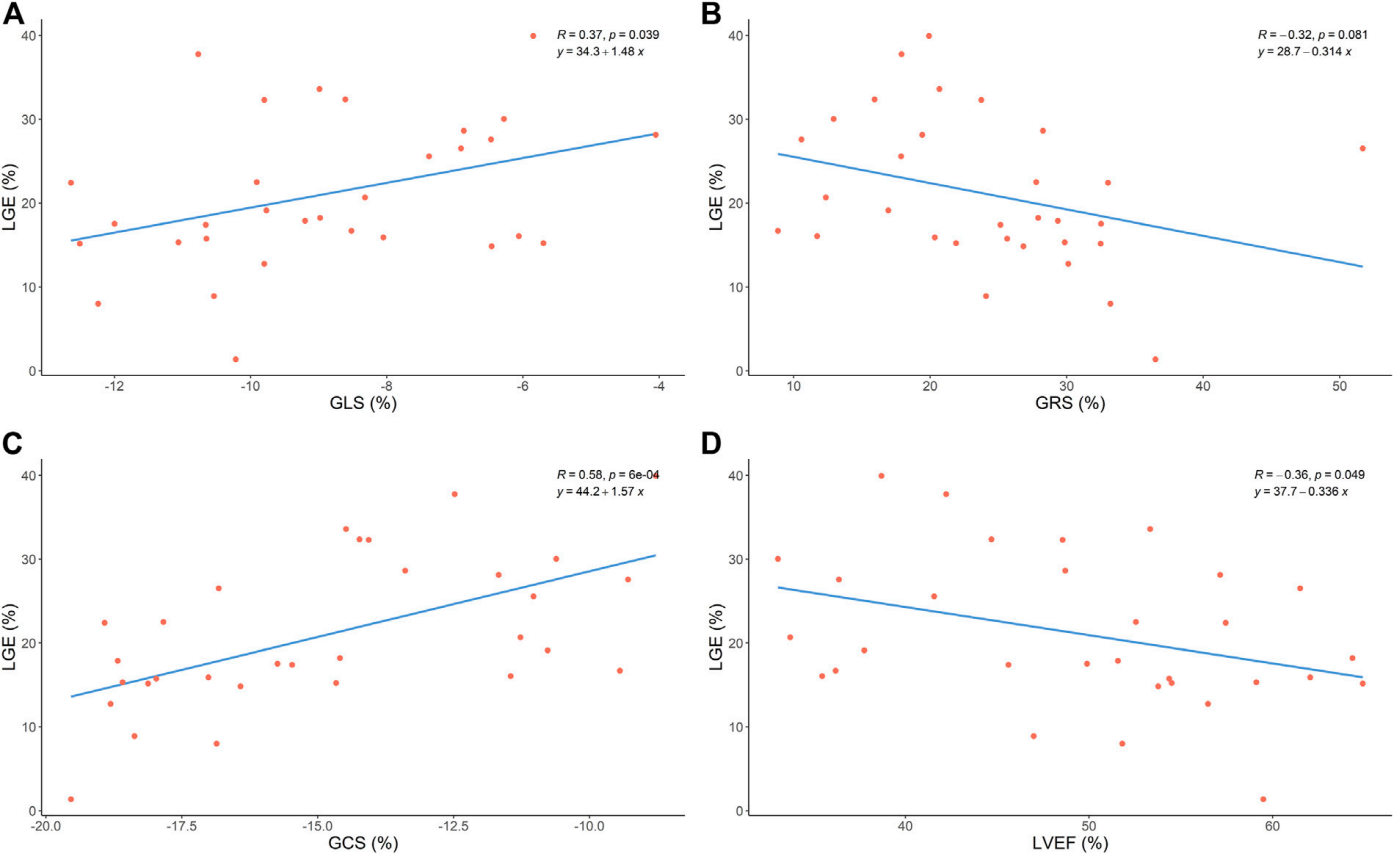
Correlation between global strain, LVEF and LGE size. GLS, global longitudinal strain; GRS, global radial strain; GCS, global circumferential strain;LVEF, left ventricular ejection fraction; LGE, late gadolinium enhancement.

As Figure 3 shows, LV GCS and GLS were associated with LGE (GCS: r = 0.58, *p* < 0.05; GLS: r = 0.37, *p* < 0.05). However, we found that LV GRS was not associated with LGE (GRS: r = −0.32, *p* > 0.05). Also, an inverse correlation was found between LVEF and LGE (r = −0.36, *p* < 0.05).

### Multivariable regression analysis for LGE size

We constructed the multivariable linear models to assess the relationship of LGE with GCS, GLS, and LVEF. We found that LGE was significantly associated with GCS (β coefficient = 2.11, std error = 0.82, *p* = 0.02) but not associated with GLS (β coefficient = −0.102, std error = 0.80, *p* = 0.90) and LVEF (β coefficient = 0.23, std error = 0.24, *p* = 0.35) (Table 3).

**TABLE 3:** Multivariable linear regression analysis for LGE size.

Variable	Beta	95% CI	*p*-value
GCS	2.11	0.42 to 3.80	0.02
GLS	−0.10	−1.76 to 1.56	0.90
LVEF	0.23	−0.27 to 0.72	0.35

LGE, late gadolinium enhancement; GCS, global peak circumferential strain; GLS, global peak longitudinal strain; LVEF, left ventricular ejection fraction.

### Receiver operating characteristic analysis for prediction of LGE size

**FIGURE 4:**
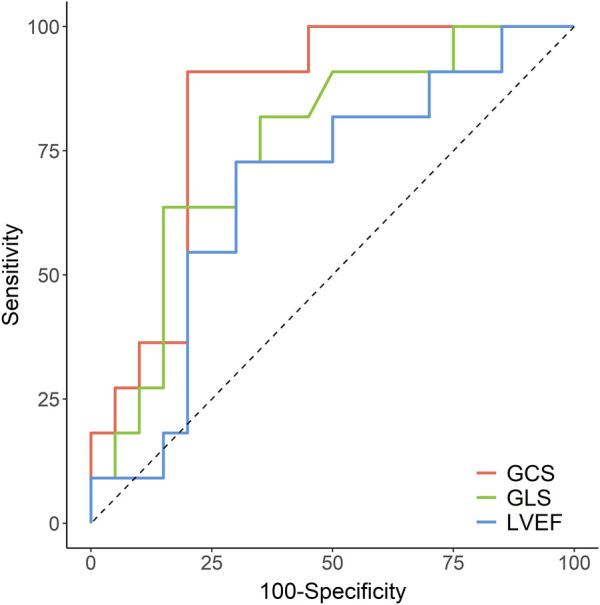
Receiver operating characteristic curves for accuracy of GCS, GLS and LVEF in the prediction of LGE>25%. GCS, global circumferential strain; GLS, global longitudinal strain; LVEF, left ventricular ejection fraction; LGE, late gadolinium enhancement.

The area under the curve (AUC) for the ability of each cardiac function parameter to correctly identify the LGE size of the STEMI patients with preserved LVEF (whether higher than 25% or not) were as follows: GCS: 0.836 [95% CI, 0.692—0.981], *p* = 0.001; GLS: 0.761 [95% CI, 0.583–0.939], *p* = 0.009, and for LVEF: 0.673 [95% CI, 0.469—0.877], *p* = 0.061 (Figure 4). The cut-off values of GCS, GLS, and LVEF were −14.54% (sensitivity: 91%, specificity: 80%), −7.72% (sensitivity: 64%, specificity: 85%), and 49.30 (sensitivity: 73%, specificity: 70%), respectively. Furthermore, the comparison of the AUC values for the assessed cardiac function parameters showed that GCS had a significantly higher diagnostic accuracy than LVEF (*p* < 0.05) but no statistical significance with GLS (*p* > 0.05).

## Discussion

In the present study, we investigated the characteristics of conventional CMR parameters and the correlation between the strain and LGE assessment by CMR-FT in STEMI patients with preserved LVEF. Our research has found that STEMI patients with preserved LVEF have impaired segmental strain, GLS, GCS, and GRS. Compared with healthy controls, STEMI patients had enlarged LVEDV, LVEDVi, LVESV, LVESVi, and LAV_pre-a_. Additionally, LVSVi and total LA EF tended to decline in patients. However, we did not find any significant differences in RVEF, RVEDV, RVEDVi, RVESV, RVESVi, or RVSVi between the two groups. GCS, GLS, and LVEF were associated with LGE size. Furthermore, GCS had superior diagnostic accuracy to GLS and LVEF in identifying myocardial infarction size in the STEMI patients with preserved LVEF. This work demonstrates that it may be possible to detect infarction size when GCS is impaired.

Although an integrated myocardial infarction emergency system allows more patients to receive timely revascularization treatment and is more likely to minimize the size of the infarcted myocardium, it is essential to note that the adult heart does not possess any regenerative capacity. Therefore, a scar is formed when the infarcted myocardium heals. As part of the healing process, alarmins released by dying cells trigger a cascade of inflammatory reactions. When fibroblasts are activated by the renin-angiotensin-aldosterone system and released by transforming growth factor-β (Sun and Weber, 1996), they become myofibroblasts and produce an extracellular matrix (Moore-Morris et al., 2014). Generally, the procedure for healing an infarct is associated with a geometric remodeling of the chamber, characterized by dilation, hypertrophy, and progressive dysfunction of viable segments (Mukherjee et al., 2003).

Our study included STEMI patients with preserved LVEF, suggesting that left ventricular systolic function may be less affected by myocardial infarction, and infarct size may be smaller. However, compared with the control group, we found that the left ventricular volume CMR parameters LVEDV, LVEDVi, LVESV, and LVESVi were significantly increased (*p* < 0.05). Several different processes may cause expansion of the infarcted segment. First, this may reduce the number of layers across the ventricular wall due to the rearrangement of bundles of cardiomyocytes, accompanied by the elongation of these cardiomyocytes. Second, through activation of matrix metalloproteinases (MMPs), the interstitial matrix may be degradable, leading to increased dilation of the chambers (Radwan et al., 2021). Third, the organization, spatial polarization, and contraction of infarct myofibroblasts may result in perturbed scar maturation, stimulating the dilation of the chambers.

Compared with the control group, we did not find significant changes in right ventricular (RV) CMR parameters in patients (*p* > 0.05), perhaps due to the patient’s left ventricular systolic function being preserved due to ventricular interdependence, resulting in little effect on the right ventricle. RV dysfunction is associated with worse in-hospital outcomes regardless of the localization of AMI and a lower 1-year survival rate (Mukherjee et al., 2003). Thus, we can infer that STEMI patients with preserved LVEF and normal right ventricular function are more likely to have better short-term outcomes.

In this study, left atrial (LA) parameters were also measured, and we found that LAEF was significantly decreased compared to the control group (*p* < 0.05). A more extensive analysis by Ledwoch and others, including 684 STEMI patients, measured LA function only by ejection fraction and found an independent association between LAEF and adverse clinical events. However, the prognostic value of LAEF was not incremental to LVEF (Ledwoch et al., 2018). Therefore, a reduction in LAEF is a predictor of adverse clinical events.

Different researchers have demonstrated that LVEF for predicting challenging clinical events post-STEMI is independent and incremental to established outcome factors such as echocardiography-based LVEF (Bodi et al., 2009; de Waha et al., 2010; de Waha et al., 2014). Additionally, LVEF is limited by reflecting only global systolic function while insufficiently conveying regional functional abnormalities (Smiseth et al., 2016; Amzulescu et al., 2019). Echocardiography showed that every subject in this study had a generally normal LVEF. Cardiologists have noted that the LVEF cannot predict the infarct size or estimate the degree of damage to its global or segmental functions. Hence, cardiologists cannot formulate treatment plans that are more appropriate and individualized, such as earlier and more appropriate use of angiotensin-converting enzyme inhibitor (ACEI), angiotensin receptor blockers (ARB), angiotensin receptor neprilysin inhibitor (ARNI), β-blockers, and sodium-dependent glucose transporters two inhibitors (SGLT-2i) (Paolisso et al., 2022). The results of our study suggest that LVEF measured by CMR in patients was significantly lower than in the control group (49.48 ± 9.5% vs. 62.88 ± 6.96%, *p* < 0.05). Nevertheless, LVEF is still critical, making it difficult for cardiologists to judge the infarction size accurately, predict outcomes, and give appropriate treatment guidance based on the parameter.

An analysis of myocardial strain evaluates deformations of the myocardium throughout the cardiac cycle, providing information on global and regional LV function (Smiseth et al., 2016; Amzulescu et al., 2019). Myocardial strain analysis is a powerful tool to quantify subtle and regional myocardial dysfunction over LVEF (Khan et al., 2016a; Mangion et al., 2017). CMR is currently considered the standard test for determining LV morphology, and the development of FT-CMR has enabled a highly accurate assessment of myocardial strain. Our analysis confirmed that several previous studies, including STEMI patients, demonstrated excellent intra- and inter-observer variability in FT-CMR (Eitel et al., 2018).

Several investigations have shown that cine-derived strain analyses can be performed with high accuracy and reproducibility after STEMI (Hor et al., 2010; Khan et al., 2015; Mangion et al., 2017). The global strain parameters derived from early CMR after STEMI have been prognostic. A study by Martin Reindl and others found that all three global strain measures (GLS, GRS, and GCS) are significantly correlated with major adverse cardiovascular events (MACE) (Reindl et al., 2019). Yu et al. reported that global strain was first impaired in anterior STEMI patients with normal LVEF (Yu et al., 2021). In our study, all global strain parameters (GLS, GCS, and GRS) and segmental strain parameters (apical, intermediate, and basal strains) except basal radial strain were significantly decreased in STEMI patients with preserved LVEF.

The results of this study are consistent with those of a previous study. Notably, our population did not define the site of myocardial infarction, indicating that global and segmental strain has decreased regardless of the location of the myocardial infarction in patients. However, the global strain had better reproducibility than the segmental strain in CMR feature-tracking (Dobrovie et al., 2019). Global strain is more accurate than segmental strain for assessing cardiac function and predicting the degree of cardiac damage. CMR is the gold standard for assessing of infarct size (IS) in STEMI patients. Accordingly, the quantification of acute IS by LGE should be performed 3—7 days after an MI (Lund et al., 2007). It has been demonstrated that acute IS quantified in this time frame is highly predictive of outcomes in STEMI patients (Larose et al., 2010). Microvascular obstruction (MVO) is recognized as a strong predictor of adverse clinical outcomes after STEMI. The index of microcirculatory resistance (IMR) provides assessment of coronary microvascular status early after primary PCI. IMR has been identified as a predictor of change in LVEF and IS after STEMI. De Maria GL et al. found a relation between IS assessed by CMR and microvascular dysfunction (De Maria et al., 2019). Therefore, IS measured in the acute phase after STEMI predicts LV remodeling and complex clinical outcomes (Lund et al., 2007; Eitel et al., 2013).

IS by LGE has been recommended as a primary CMR endpoint measure in experimental and clinical trials (Ibanez et al., 2019). For the relationship between LGE and global strain after myocardial infarction, there is only a moderate correlation between global strain and infarction size when assessed early post-STEMI. A study conducted by Khan et al. included 24 patients (8% with anterior myocardial infarctions) with STEMI who underwent CMR on average 2.2 days after PCI showed that there was a moderate correlation between the total IS and GLS, GCS, and GRS, which were significantly lower in segments with infarct than in segments without (Khan et al., 2015). A study by Yu et al. (Yu et al., 2021) included 129 acute anterior myocardial infarction patients who underwent CMR 1 month after surgery and showed a strong association between strain parameters and LGE (GRS: r = 0.65; GCS: r = 0.69; GLS: r = 0.61).

Unlike our study, previous ones did not delineate study populations based on LVEF, whereas we included patients with preserved LVEF. In addition, our patients underwent CMR up to 14 days after PCI, and the site of myocardial infarction included the anterior and inferior-posterior walls. Our results showed that there was a moderate correlation between GCS and LGE (r = 0.58, *p* < 0.05) and a weak correlation between GLS and LGE (r = 0.37, *p* < 0.05), but we found no correlation between GRS and LGE (*p* > 0.05). In the multivariate model, we found that the LGE size was significantly associated with GCS (β coefficient = 2.110, *p* = 0.016) but was not associated with GLS (β coefficient = −0.102, *p* = 0.900) or LVEF (β coefficient = 0.227, *p* = 0.354).

Consistent with the above findings, GCS was correlated with the LGE size. Khan et al. also found a moderate correlation between GCS and IS but did not find a correlation between GLS and IS (Khan et al., 2015), possibly due to the small proportion of patients with anterior myocardial infarction that were included in their study as longitudinal myofibers are typically located in the mid-endocardium, and particularly in the anterior wall, which has a more significant impact on cardiac function. Yu et al. (Yu et al., 2021) found that GCS, GLS, and GRS were associated with LGE because they enrolled patients with anterior myocardial infarction regardless of LVEF. Larger infarcted myocardium in the anterior wall region may carry more substantial damage in both longitudinal myocardial fibers located in the mid-endocardium and circumferential myocardial fibers on the epicardial side. Therefore, GCS, GLS, and GRS are all correlated with LGE.

Based on the studies mentioned above, we believe there is a correlation between GCS and LGE in STEMI patients. A possible pathophysiological explanation is that the subendocardium is more susceptible to ischemic injury than the mid-myocardium and subepicardium. A “wavefront of necrosis” is, therefore, widely accepted as a mechanism of cardiomyocyte death that occurs during the progression of ischemic injury from the subendocardium to the subepicardium due to the increased duration of the insult (Reimer et al., 1977). In addition, circumferential myofibers are typically located on the epicardial aspect of the heart, so the infarct area spreads from the endocardium to the epicardium with prolonged ischemia time, and circumferential fibers suffer more damage as the infarct size expands (Bogaert et al., 2000).

Furthermore, the circumferential strain has a more excellent discriminative value for assessing the transmural extent of infarction in patients with recent MI (Koos et al., 2013). The circumferential strain rate has incremental prognostic use in predicting LV functional recovery in the longer term after an acute STEMI (Neizel et al., 2010). Moreover, our study found a weak correlation between GLS and LGE, perhaps because the selected patients had an approximately normal LVEF or only mild impairment. Longitudinal myofibers are typically located in the mid-endocardium, and the “wavefront phenomenon” describes ischemic myocardial injury starting at the subendocardial layers. Research has shown that GLS is a modest predictor of adverse remodeling of the LV and provides strong validity for the prediction of MACE post-STEMI. As a result, GLS may be slightly less affected than GCS in STEMI patients with preserved LVEF and only weakly correlated with LGE, suggesting that our study population may have a relatively good prognosis with a lower incidence of MACE than the general population with STEMI. GRS represents radially directed myocardial deformation toward the center of the LV cavity and indicates the LV thickening and thinning motion during the cardiac cycle. In contrast to the findings of Shiqin Yu, we found no significant correlation between GRS and LGE, possibly because about two-thirds of the patients in their study had decreased LVEF. It is possible that different infarct sizes and locations in patients can affect GRS differently.

A previous study reported that >25% of LGE extent was hard to recover and implied adverse outcomes despite successful revascularization (Kim et al., 2000). Our study showed that GCS discriminated well for LGE >25% (AUC: 0.836 [95% CI, 0.692—0.981]). However, GLS is less effective in predicting LGE >25% (AUC: 0.761 [95% CI, 0.583—0.939]). LVEF is not an ideal indicator of LGE >25% [AUC: 0.673 (95% CI, 0.469—0.877)]. Khan et al. found that accuracy in predicting segmental transmural LGE was greatest for FT-derived GCS (AUC: 0.772) (Khan et al., 2015). In patients with acute anterior myocardial infarction, Yu et al. found that segmental CS was more valuable in predicting segmental LGE >50% (AUC: 0.903) (Yu et al., 2021).

Therefore, we believe that GCS may effectively predict infarct size in STEMI patients with preserved LVEF. Although GLS is a better predictor of MACE, it is less accurate at predicting infarct size than GCS. Even though LVEF is one of the most commonly used clinical indicators to reflect cardiac function, it does not help predict infarct size. In a group of patients referred for CMR (11% with coronary artery disease) (Mordi et al., 2015), tagging-derived GCS was a multivariate predictor of MACE. Some patients develop renal impairment after acute MI, which makes them ineligible for gadolinium-contrast imaging. It is also possible to use strain imaging when gadolinium-contrast medium cannot be tolerated to assess infarct size. The strain provides more direct information on regional and global LV function in patients with acute MI than LVEF. Cardiologists can use CMR to measure GCS early after STEMI to estimate the infarct size and optimize drug therapy more efficiently. In addition, the myocardial strain has the potential to provide biomarkers that are predictive of LV recovery after STEMI. Patients with preserved LVEF may benefit from strain measurement in assessing treatment effectiveness predicated on improved precision and accuracy over LVEF. Using strain imaging in patient follow-up may facilitate the evaluation of infarct size, scarring, myocardial fibrosis, and MACE and allow cardiologists to adjust medications such as antiplatelet therapy (Khan et al., 2016b; Cimmino et al., 2020), angiotensin-converting enzyme inhibitor (ACEI), angiotensin receptor blockers (ARB), angiotensin receptor neprilysin inhibitor (ARNI), β-blockers and SGLT-2i (Paolisso et al., 2022) more precisely.

This study had several limitations. First, the sample size was relatively small. Second, this study was a single-center, retrospective study based on the preferred time point for assessing late gadolinium enhancement imaging. CMR scans were performed up to 14 days after MI, but it is not sure that this time point was optimal for the CMR measurements. Third, because the study focused on STEMI patients with preserved LVEF, extrapolating the findings to STEMI patients who had decreased LVEF or non-STEMI patients was limited. Moreover, we included patients with preserved LVEF based on echocardiographic findings, which were likely to be affected by equipment and operator factors. Furthermore, we could not perform predictive analysis on the relationship between strain, LGE, and prognosis due to a lack of follow-up for many patients. There may be a need for a more comprehensive prospective study with a more significant number of participants.

In conclusion, global and segmental strain were impaired in STEMI patients with preserved LVEF treated by contemporary primary percutaneous coronary intervention. GCS and LGE were correlated, and GCS, as determined by CMR, emerged as a strong and independent predictor of infarction size. Moreover, GCS had superior diagnostic accuracy to GLS and LVEF for predicting myocardial infarction size. Consequently, multiparametric CMR imaging (including the strain) has an emerging role in infarction size prediction in post-STEMI patients that warrants further research.

## Data Availability

The original contributions presented in the study are included in the article/Supplementary Material, further inquiries can be directed to the corresponding authors.
